# Characterization of RmlABCD Enzymes from Marine Bacteria and Efficient Synthesis of dTDP-L-Rhamnose

**DOI:** 10.3390/microorganisms14051070

**Published:** 2026-05-09

**Authors:** Jinghua Li, Rui Geng, Junfeng Chen, Wei Wang, Shengbo Shi, Longyu Fang, Yuanyuan Wang, Mingchun Lin, Yanru Si, Lujiang Hao

**Affiliations:** 1 School of Bioengineering, Qilu University of Technology (Shandong Academy of Sciences), Jinan 250353, China; 10431230807@stu.qlu.edu.cn (J.L.); 10431230774@stu.qlu.edu.cn (R.G.); 10431240800@stu.qlu.edu.cn (J.C.); 10431230730@stu.qlu.edu.cn (S.S.); 10431230825@stu.qlu.edu.cn (L.F.); 10431230832@stu.qlu.edu.cn (Y.W.); 10431240798@stu.qlu.edu.cn (M.L.); 10431240837@stu.qlu.edu.cn (Y.S.); 2 Jiangsu Key Laboratory for Microbes and Functional Genomics, Engineering and Technology Research Center for Microbiology, College of Life Sciences, Nanjing Normal University, Nanjing 210023, China; 241201017@njnu.edu.cn

**Keywords:** *Pseudoalteromonas agarivorans*, rhamnose biosynthesis, marine enzymes, dTDP-L-rhamnose, biosynthesis

## Abstract

dTDP-L-rhamnose (Deoxythymidine diphospho-L-rhamnose) is a crucial active sugar nucleotide that serves as the key glycosyl donor for the synthesis of rhamnose-containing polysaccharides in bacteria, holding broad application potential in pathogen-associated molecular mimicry and vaccine development. In this study, the rhamnose synthase gene cluster (*Pa*-RmlABCD) was successfully cloned for the first time from the marine bacterium *Pseudoalteromonas agarivorans* Hao 2018. Four key enzymes—Glc-1-P thymidylyltransferase (*Pa*-RmlA), dTDP-glucose-4,6-dehydratase (*Pa*-RmlB), dTDP-4-keto-6-deoxyglucose 3,5-epimerase (*Pa*-RmlC), and dTDP-4-keto-rhamnose reductase (*Pa*-RmlD)—were heterologously expressed in *Escherichia coli*. A one-pot four-enzyme synthesis system was constructed, and the successful synthesis of dTDP-L-rhamnose was verified by Q Exactive Focus. After correction for recovery (92% ± 2%), the actual yield reached 3.47 mg/L with a conversion rate of 53.4% ± 1.1%. Combined with bioinformatics analysis, tertiary structure modeling, and molecular docking simulations, the sequence characteristics, substrate binding modes, and catalytic mechanisms of *Pa*-RmlABCD were systematically elucidated. By characterizing the marine-derived *Pa*-RmlABCD system and achieving efficient one-pot synthesis, this work opens up a new avenue for the sustainable production of dTDP-L-rhamnose, with the potential to alleviate the current industrial supply constraints.

## 1. Introduction

L-Rhamnose, a naturally occurring L-deoxyhexose, is widely distributed in both plant and microbial systems [1], where it serves as a key structural component of lipopolysaccharide (LPS) O-antigens and bioactive extracellular polysaccharides [2]. Compounds incorporating this monosaccharide have been shown to possess potent antitumor activity [3]; moreover, rhamnosylation modification can improve target specificity and biocompatibility [4]. Significantly, microbial rhamnose biosynthesis pathways have emerged as potential therapeutic targets for antibacterial applications [4]. As a result, rhamnose-containing molecules are considered core precursors for the development of vaccines, chemotherapeutics, and antimicrobial agents [5,6], generating a substantial and largely unmet demand for their efficient, large-scale production and establishing rhamnose biosynthesis as a global research priority [7].

Despite increasing market demand for rhamnose-derived pharmaceuticals, vaccines, and functional foods, industrial implementation is severely constrained by a critical supply bottleneck: the dTDP-rhamnose synthesis genes dTDP-L-rhamnose [8]. Conventional chemical synthesis suffers from lengthy reaction sequences, poor selectivity, and inconsistent purity, while traditional biocatalytic approaches rely on excessively costly nucleotide diphosphate sugars (e.g., dTDP-D-glucose) and require tedious, multi-step purification procedures [9]. Consequently, production costs remain extremely high—with commercially available products priced at thousands of yuan per gram—and yields are insufficient to support downstream bioprocesses [10]. Although the canonical four-enzyme pathway (RmlA–D) responsible for assembling dTDP-L-rhamnose was initially characterized in terrestrial bacteria during the mid-20th century [11], existing heterologous platforms (such as the *Ss*-rmlABCD system from *Saccharothrix syringae* CGMCC 4.1716 or stepwise strategies employing *Streptococcus pneumoniae* 23F) are still economically unfavorable due to inherent inefficiencies, low stability of crude enzymes, and the necessity for intermediate isolation [9,12,13,14].

Accordingly, the field faces a dual challenge of enhancing catalytic efficiency while significantly lowering manufacturing costs [15,16]. Enzymes derived from terrestrial bacteria appear to have reached a performance plateau, with protein engineering yielding only marginal gains. At the same time, industrial requirements for >95% product purity and a >60% reduction in production costs remain difficult to achieve using legacy technologies, which are hampered by suboptimal reaction kinetics and low product recovery rates [17,18]. Although recent efforts have explored the use of low-cost substrates (e.g., dTMP or sucrose) in multi-enzyme cascade systems, these approaches do not fully address the fundamental economic limitations, and the technology readiness level (TRL) is still inadequate for industrial deployment [19,20]. Notably, previous studies have focused almost exclusively on terrestrial microorganisms, leaving the biosynthetic capabilities of marine bacteria completely unexplored [21,22]. Considering the extremophilic conditions of marine habitats—marked by high salinity, low temperature, and elevated hydrostatic pressure—marine microbes are likely to possess distinctive metabolic pathways and robust enzymatic systems [23,24]. Such biocatalysts hold untapped potential for superior stability and catalytic efficiency, offering a promising avenue for overcoming current technological barriers in industrial rhamnose biosynthesis.

In previous work, we isolated and identified the marine bacterium *Pseudoalteromonas agarivorans* Hao 2018 (*P. agarivorans* Hao 2018) from the microbial biofilm on the surface of *Haliotis discus hannai* (wrinkled abalone) seedlings [25,26,27]. This Gram-negative marine strain has been reported to produce biologically active exopolysaccharides, and several carbohydrate metabolism related enzymes have been characterized in this strain. Using transcriptome analysis (DEGs), qRT-PCR verification, and structure activity relationship analysis of exopolysaccharides, the gene cluster responsible for dTDP-L-rhamnose biosynthesis was successfully identified. Existing studies have mostly focused on the structural characterization and activity evaluation of extracellular polysaccharides in marine bacteria, and there is a lack of systematic bioinformatics analysis and experimental validation of synthetic genes, so this study aims to make up for this deficiency.

The present study aimed to clone and heterologously express the *RmlABCD* gene cluster from marine *P. agarivorans* Hao 2018, characterize the enzymatic properties and catalytic mechanism of the four recombinant enzymes, and further construct an efficient one-pot four-enzyme catalytic system for the biosynthesis of dTDP-L-rhamnose. This work intends to provide a novel marine enzyme resource and a green synthetic route for the high-value production of glycosyl nucleotide compounds.

## 2. Materials and Methods

### 2.1. Chemicals and Strains

Glc-1-P was purchased from Sichuan Weikeqi Biotechnology Co., Ltd. (Chengdu, China), dTTP was acquired from Beyotime Biotechnology (Shanghai, China), NAD^+^ and NADH were obtained from MedChemExpress (MCE) (Shanghai, China), NADP^+^ was sourced from MedBio (Shanghai, China), NADPH was procured from Glpbio (Montclair, CA, USA), DTT was purchased from Solarbio (Beijing, China), YIPP was acquired from Beyotime Biotechnology (Shanghai, China), Restriction endonucleases (NdeI, BamHI, SacI, NotI) were obtained from Yu Gong Life Science (Lianyungang, China), PBS (phosphate-buffered saline), imidazole, and plasmids pET-22b/pET-28a were purchased from Tianjingsha Biotechnology (Beijing, China).

### 2.2. Strains and Culture Conditions

The bacterial strain *P. agarivorans* Hao 2018 (preservation number CGMCC No. 26272) was maintained in our laboratory and cultured under optimized conditions. The seed culture was grown in Zobell 2216E medium (containing 2.0 g/L yeast extract, 5.0 g/L peptone, and 35.0 g/L marine salts, pH adjusted to 7.6–7.8). Solid medium was prepared by adding 17 g/L agar to the liquid Zobell 2216E medium. The strain was cultured at 25 °C with shaking at 180 rpm for 8–12 h in a constant-temperature incubator shaker. For molecular cloning, *E. coli* DH5α (purchased from Vazyme Biotech, Nanjing, China) was used. For protein expression, *E. coli* BL21 (also from Vazyme Biotech, Nanjing, China) was cultured in Luria–Bertani (LB) medium at 37 °C. When required, 50 μg/mL ampicillin (Sangon Biotech, Shanghai, China) was added for plasmid maintenance.

### 2.3. Cloning and Heterologous Expression of Pa-RmlABCD Genes

The four genes *Pa*-RmlA, *Pa*-RmlB, *Pa*-RmlC, and *Pa*-RmlD from *P. agarivorans* Hao 2018 were amplified using four pairs of specific primers designed based on the target coding sequences (Table S1). PCR amplification was performed using the genomic DNA of P. agarivorans Hao 2018 (GenBank assembly: GCA_003668795.1) as the template. The corresponding GenBank accession numbers are as follows: *Pa*-RmlA, CP033065.1; *Pa*-RmlB, CP033065.1; *Pa*-RmlC, CP033065.1; *Pa*-RmlD, CP033065.1. Primer 5.0 software was used to design primers for the four genes and the gene cluster. Restriction enzyme sites SacI (GAGCTC), NotI (GCGGCCGC), NdeI (CATATG), and BamHI (GGATCC) were introduced into the forward and reverse primers, respectively (Table S1). Standard molecular genetic engineering techniques were used throughout this study.

The purified PCR products were cloned into the pET-22b vector by homologous recombination, with a C-terminal hexahistidine (6 × His) tag fused to each gene. The correctly constructed recombinant plasmids were confirmed by DNA sequencing and then transformed into *E. coli* BL21(DE3) for heterologous protein expression. The recombinant expression vectors pET16b-*Pa*RmlA, pET16b-*Pa*RmlB, pET16b-*Pa*RmlC, and pET28a-PaRmlD were successfully constructed (Figure S1).

The transformed cells were cultured in LB medium supplemented with 50 μg/mL ampicillin at 37 °C with shaking at 200 rpm. When the OD_600_ reached 0.6–0.8, protein expression was induced by adding 0.5 mM IPTG, followed by incubation at 16 °C for 12 h. The cells were harvested by centrifugation at 8000 rpm and 4 °C, then disrupted by ultrasonication. After centrifugation, the supernatant containing soluble recombinant enzymes was purified by Ni-NTA affinity chromatography. The target proteins were eluted with an imidazole-containing buffer and further desalted and concentrated using a 10 kDa ultrafiltration device. Protein concentrations were determined using a BCA Protein Quantification Kit.

The molecular weights of *Pa*-RmlA, *Pa*-RmlB, *Pa*-RmlC, and *Pa*-RmlD were estimated as 33.6 kDa, 40.5 kDa, 20.6 kDa, and 31.8 kDa, respectively. SDS-PAGE was performed using a 12% (*w*/*v*) gel, and proteins were visualized by Coomassie Brilliant Blue (CBB) staining. NcmColor pre-stained protein molecular weight markers were used to estimate protein sizes.

### 2.4. Bioinformatics Analysis

The comprehensive bioinformatics analysis of the dTDP-rhamnose synthesis genes (*Pa*-RmlA-D) from *P. agarivorans* Hao 2018 was performed using an integrated computational approach. The molecular weights, isoelectric points, and estimated half-lives of the four encoded enzymes were predicted using the ProtParam tool (https://www.expasy.org/, accessed on 10 March 2025), while their hydrophobicity profiles were analyzed through ProtScale (https://web.expasy.org/protscale/, accessed on 15 March 2025) to evaluate hydrophilic and hydrophobic characteristics. Potential signal peptides were identified using SignalP (https://services.healthtech.dtu.dk/services/SignalP-4.1/, accessed on 18 March 2025), and subcellular localization predictions were conducted with Cell-PLoc 2.0 (http://www.csbio.sjtu.edu.cn/, accessed on 18 March 2025). For evolutionary analysis, multiple sequence alignment of homologous enzymes from different species was performed using Clustal Omega (https://www.ebi.ac.uk/services, accessed on 26 March 2025), followed by phylogenetic tree construction with MEGA X software (version 10.2.6).

The three-dimensional structures of the enzymes were predicted using AlphaFold3 (https://alphafold.com/, accessed on 27 March 2025), with subsequent format conversion accomplished by Open Babel 3.1.1. Model quality was rigorously assessed through the SAVES server (https://saves.mbi.ucla.edu/, accessed on 27 March 2025), which provided various validation metrics. Molecular docking studies were carried out using AutoDock Tools 1.5.7 (https://autodock.scripps.edu/, accessed on 27 March 2025), and the resulting protein-ligand interactions were visualized and analyzed with PyMOL 2.5 (https://pymol.org/2/, accessed on 28 March 2025) and the Protein-Ligand Interaction Profiler (https://plip-tool.biotec.tu-dresden.de/plip-web/plip/index, accessed on 28 March 2025), respectively. This multi-faceted computational strategy enabled systematic characterization of the structural and functional properties of the dTDP-rhamnose biosynthetic enzymes.

### 2.5. Functional Characterization of Pa-RmlABCD

An InertSustain C18 column (4.6 × 250 mm) was used for high performance liquid chromatography tandem mass spectrometry (HPLC) analysis. The mobile phase consisted of 0.1 mM ammonium acetate, with a flow rate of 0.8 mL/min. A ultraviolet (UV) detector was employed at a wavelength of 260 nm, and the detection temperature was maintained at 30 °C. For each HPLC run, 200 μL of the sample was injected.

A Q Exactive Focus hybrid quadrupole-orbitrap mass spectrometer was utilized, combining the high separation efficiency of ultra-high-performance liquid chromatography (UHPLC) with the high resolution and accurate mass analysis capabilities of the quadrupole-orbitrap system. This setup enabled effective separation and identification of carbohydrate compounds in complex samples via mass spectrometry (MS). The mobile phases were: Phase A, 0.1% formic acid in water; Phase B, 0.1% formic acid in acetonitrile. Samples were injected using a flow injection pump at a flow rate of 0.3 mL/min, with an injection volume of 5 μL. The resolution was maintained at 70,000 FWHM, and the scan range was set to *m*/*z* 50–1000 in negative ion mode. The electrospray voltage was −4.5 kV. Full-scan MS was acquired at a resolution of 70,000, and data-dependent tandem MS scans were performed at a resolution of 17,500. The automatic gain control (AGC) targets were 5 × 10^6^ for full scans and 2 × 10^6^ for MS/MS scans.

The gradient elution program was optimized to separate dTDP-L-rhamnose from impurities through a multi-stage mobile phase adjustment. Initially (0.0 min), the system started with 90% Phase A (0.1% formic acid in water) and 10% Phase B (0.1% formic acid in acetonitrile) to retain medium-polarity dTDP-L-rhamnose while preventing co-elution with strongly polar impurities. Over the first 3.0 min, Phase A linearly decreased to 70% and Phase B increased to 30% for preliminary separation of strongly polar contaminants. From 3.0 to 6.0 min, Phase A further decreased to 50% and Phase B increased to 50%, gently eluting dTDP-L-rhamnose and achieving baseline separation from structurally similar intermediates. A rapid gradient (6.0–9.0 min) then decreased Phase A to 5% and increased Phase B to 95% to efficiently remove weakly polar impurities. This composition was maintained for 3 min (9.0–12.0 min) to ensure complete impurity clearance. The system rapidly returned to initial conditions (90% A/10% B) over 0.5 min (12.0–12.5 min) to prevent column pressure fluctuations, followed by 2.5 min of equilibration (12.5–15.0 min) at the starting ratio. This method combines HPLC separation efficiency with high-resolution mass spectrometry capabilities for accurate identification.

### 2.6. Enzymatic Activity Assay of Pa-RmlABCD

The activity of *Pa*-RmlA was determined using a pyrophosphate-coupled colorimetric assay with minor modifications [28]. A 100 μL reaction mixture containing 40 mM Tris-HCl buffer (pH 8.0), 5.0 mM dTTP, 5.0 mM Glc-1-P, 10 mM MgCl_2_, 100 μg/mL *Pa*-RmlA, and 2 U/mL YIPP was incubated at 37 °C for 5 min, then mixed with 100 μL of malachite green reagent (containing 0.03% (*w*/*v*) malachite green, 0.2% (*w*/*v*) ammonium molybdate, and 0.05% (*v*/*v*) Triton X-100) to terminate the reaction. For time-course analysis, 50 μL of malachite green coloring solution (composed of 0.35‰ (*w*/*v*) malachite green, 0.5‰ (*v*/*v*) Triton X-100, 2.5‰ (*w*/*v*) ammonium molybdate, and 0.7 M HCl as the solvent) was added at 10, 20, 30, 40, 60, 90, or 120 min of incubation at 37 °C to stop the reaction. After further incubation at 37 °C for 5 min, the absorbance at 630 nm was measured using a microplate reader. The amount of released pyrophosphate (PPi) in the reaction was calculated using a standard curve of OD_630_ values versus PPi concentrations. One unit of *Pa*-RmlA activity was defined as the amount of *Pa*-RmlA that catalyzes the generation of 1 μmol of PPi per minute under the experimental conditions. The experiment (Table S2) included a control group (without substrate), single-substrate groups (with only dTTP or Glc-1-P), and double-substrate groups (with both dTTP and Glc-1-P).

The activity of *Pa*-RmlB was determined using a post-colorimetric assay based on the detection of ketone group formation [29,30]. A 100 μL reaction mixture containing 40 mM Tris-HCl buffer (pH 7.4), 5.0 mM dTDP-d-glucose, and 100 μg/mL *Pa*-RmlB was incubated at 37 °C for 5 min, then mixed with 10 μL of 1 M NaOH solution and incubated at room temperature for 10 min. The OD_320_ was measured using a microplate reader, and the amount of dTDP-4-keto-6-deoxyglucose was calculated using a standard curve based on OD_320_ values. One unit of *Pa*-RmlB activity was defined as the amount of *Pa*-RmlB that catalyzes the formation of 1 μmol of dTDP-4-keto-6-deoxyglucose per minute under the experimental conditions (Table S3).

The product of the third step in the dTDP-L-rhamnose biosynthetic pathway is dTDP-4-keto-L-rhamnose, which is present in low quantities and is unstable. Under conditions where dTTP and Glc-1-P were used as substrates, with the addition of the coenzyme NADH and *Pa-*RmlABCD), NADH and substrates are theoretically consumed to generate NAD^+^ and dTDP-rhamnose. Since NADH exhibits a specific absorption peak at 340 nm, changes in OD_340_ values were measured to assess the activity of *Pa*-RmlC and *Pa*-RmlD. The experiment (Table S4) included a control group (without enzymes) and experimental groups (*Pa*-RmlA/B, *Pa*-RmlA/B/C, or *Pa*-RmlABCD). Under conditions of 37 °C and pH 7.5, the absorbance at 340 nm was measured using a microplate reader after incubation for 10, 20, 30, 40, 60, 90, or 120 min. One unit of *Pa*-RmlC activity was defined as the amount of enzyme required to catalyze the formation of 1 μmol of dTDP-4-keto-L-rhamnose per minute under optimal reaction conditions. One unit of *Pa*-RmlD activity was defined as the amount of enzyme required to oxidize 1 μmol of NAD(P)H (or generate 1 μmol of dTDP-L-rhamnose) per minute under optimal reaction conditions.

### 2.7. Biochemical Characterization of Pa-RmlA

To accurately measure the activity of *Pa*-RmlA, it is critical to ensure that the reaction proceeds within the initial velocity range. This requires determining the linear ranges of enzyme concentration and reaction time for the enzymatic reaction. To identify the linear enzyme concentration range, *Pa*-RmlA protein concentrations were varied (0.835 μg/mL, 1.669 μg/mL, 2.504 μg/mL, 4.174 μg/mL, 5.843 μg/mL, 8.348 μg/mL), with other reaction conditions added according to Table S2. The absorbance at 630 nm was measured using a microplate reader after color development following incubation at 37 °C for 3 min, 9 min, and 18 min to determine the linear enzyme concentration range. For the linear time range, *Pa*-RmlA was added at concentrations of 0.835 μg/mL, 1.669 μg/mL, 4.174 μg/mL, and 8.348 μg/mL. The reaction mixture was incubated at 37 °C for 1–6 min, and the absorbance at 630 nm was measured after color development to determine the linear time range of the enzymatic reaction.

To investigate the effect of temperature on enzymatic activity, reactions were conducted at temperatures ranging from 10 °C to 90 °C (with a 10 °C incremental gradient). The OD_630_ was measured 5 min after color development for each temperature. The reaction with the highest OD_630_ value was used as the reference (with *Pa*-RmlA activity under these conditions defined as 100%), and the relative enzyme activity at other temperatures was calculated to determine the temperature dependence of the reaction. To assess the effect of pH, enzymatic reactions were performed in buffers with pH values ranging from 5.5 to 10.To evaluate the effect of Mg^2+^, reactions were conducted with varying concentrations of Mg^2+^ (0, 5, 10, 20, and 40 mM) added to the reaction system.

For the kinetic study of *Pa*-RmlA with respect to dTTP, reactions were performed within the initial velocity range under the previously determined optimal temperature, optimal pH, and Mg^2+^ concentration, with excess Glc-1-P ensured. Reactions were conducted by varying dTTP concentrations. After incubation at 50 °C for 3 min, 50 μL of malachite green color reagent was added to terminate the reaction, followed by a 5 min incubation. The absorbance at 630 nm was measured using a microplate reader to perform kinetic analysis of *Pa*-RmlA with dTTP, and the Michaelis constant (*K_m_*) and turnover number (*k_cat_*) were calculated.

For the kinetic study of *Pa*-RmlA with respect to Glc-1-P, dTTP was provided in excess, and reactions were conducted by varying Glc-1-P concentrations. All other steps were identical to those used for the dTTP kinetic analysis, and the *K_m_* and *k_cat_* values for Gla-1-P were calculated.

### 2.8. One-Pot Synthesis of dTDP-L-Rhamnose

Purified *Pa*-RmlABCD enzymes, along with corresponding substrates and buffer, were added to a single reaction vessel. Specifically, the four purified enzymes were introduced into a 50 mM Tris-HCl buffer (pH 7.5) containing 10% (*v*/*v*) glycerol, 1 mM DTT, 2 U/mL YIPP, 5 mM MgCl_2_, 1 mM dTTP, and 1 mM Glc-1-P, with a total reaction volume of 0.5 mL. The mixture was incubated at 37 °C for 30 min, after which 3 volumes of methanol were added and thoroughly vortexed. The reaction solution was then transferred to a 10 kDa ultrafiltration tube, centrifuged at 8000 rpm for 30 min to remove proteins, and the flow-through containing dTDP-L-rhamnose was collected.

### 2.9. Statistical Analysis

All experimental data were derived from a minimum of three independent replicate experiments (n ≥ 3). Results are expressed as the mean ± standard deviation (SD). Enzyme kinetic parameters (*K_m_* and *V_max_*) for *Pa*-RmlA were determined by fitting the initial velocity data to the Michaelis-Menten equation using nonlinear regression analysis implemented in GraphPad Prism software (Version 9.0.0, GraphPad Software, San Diego, CA, USA). For product quantification via HPLC, the standard curve was generated using linear regression analysis based on the least-squares method; a standard curve was considered valid and used for quantification only if the coefficient of determination (R^2^) exceeded 0.99. Formal significance testing for comparing group differences was not performed, as the primary focus of this study was the establishment and validation of the synthetic methodology rather than comparative hypothesis testing. Should such comparisons be warranted in future studies, a *t*-test or one-way ANOVA with a significance threshold of *p* < 0.05 would be appropriate. All statistical treatments were applied under the assumption that the data approximated a normal distribution.

## 3. Results

### 3.1. Gene and Protein Sequence Analysis of Pa-RmlA, Pa-RmlB, Pa-RmlC, and Pa-RmlD

**Figure 1:**
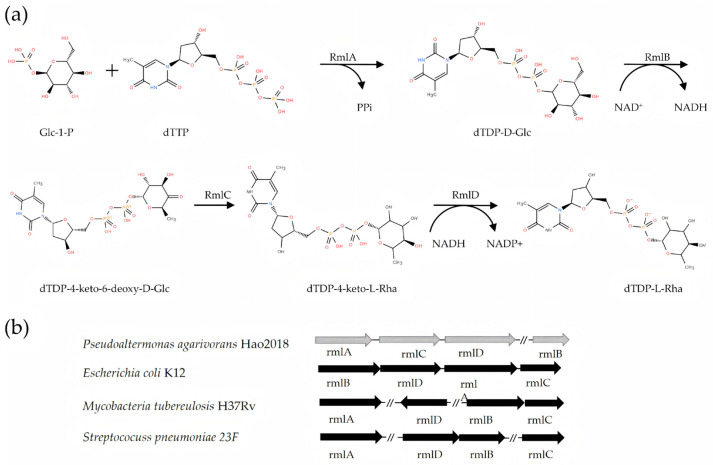
Biosynthesis of dTDP-L-Rhamnose and genetic organization of the *RmlABCD* gene cluster. (**a**) Schematic representation of the four-step biosynthetic pathway of dTDP-L-rhamnose from Glc-1-P and dTTP, sequentially catalyzed by *Pa-RmlA*, *Pa-RmlB*, *Pa-RmlC*, and *Pa-RmlD*. The cofactors involved in each reaction are also shown. (**b**) Genomic organization of the RmlABCD gene cluster in *P. agarivorans* Hao 2018, in comparison with the gene clusters from *Escherichia coli* K12, *Mycobacterium tuberculosis* H37Rv, and *Streptococcus pneumoniae* 23F. Arrows indicate the transcriptional direction of each gene.

Genomic organization comparison with model strains including *Escherichia coli* K12, *Mycobacterium tuberculosi*s H37Rv, and *Streptococcus pneumoniae* 23F revealed that the *Pa-rmlABCD* gene cluster exhibited a unique arrangement: *RmlA*–*RmlC*–*RmlD*–*RmlB* (Figure 1).

**Figure 2:**
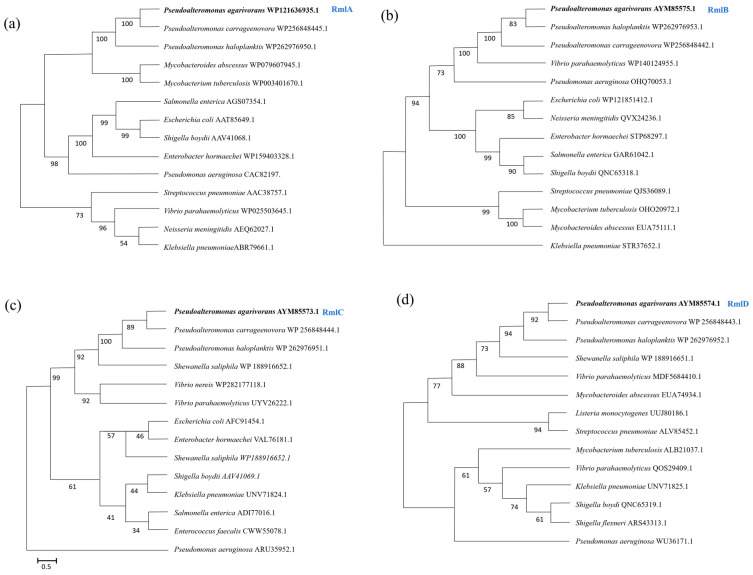
Phylogenetic relationships based on *Pa*-RmlA-D amino acid sequences from different species. (**a**) Phylogenetic tree of Pa-RmlA amino acid sequences, (**b**) Phylogenetic tree of Pa-RmlB amino acid sequences, (**c**) Phylogenetic tree of Pa-RmlC amino acid sequences, (**d**) Phylogenetic tree of Pa-RmlD amino acid sequences. Phylogenetic trees were constructed using MEGA X software with the neighbor-joining (NJ) method. Bootstrap values were calculated with 1000 replicates.

Phylogenetic analysis showed that the amino acid sequences of *Pa*-RmlABCD were highly conserved and clustered most closely with marine *Pseudoalteromonas* species, forming an independent evolutionary clade (Figure 2). Multiple sequence alignment indicated that all *Pa*-RmlABCD enzymes contained the highly conserved core functional motifs of the Rml family. *Pa*-RmlA harbored nucleotidyltransferase-specific motifs (GXGT/SRLXPXTX4K, LGDGX4) and the Mg^2+^-binding catalytic motif DTG. *Pa*-RmlB contained typical short-chain dehydrogenase/reductase (SDR) signatures (GS/GAGFIGAN, HXAAESHVDRS). *Pa*-RmlC possessed conserved epimerase motifs (DXRGFMETFR, FVQENHSKS). *Pa*-RmlD carried the reductase-specific motif TGXG/NVGQLG.

**Figure 3:**
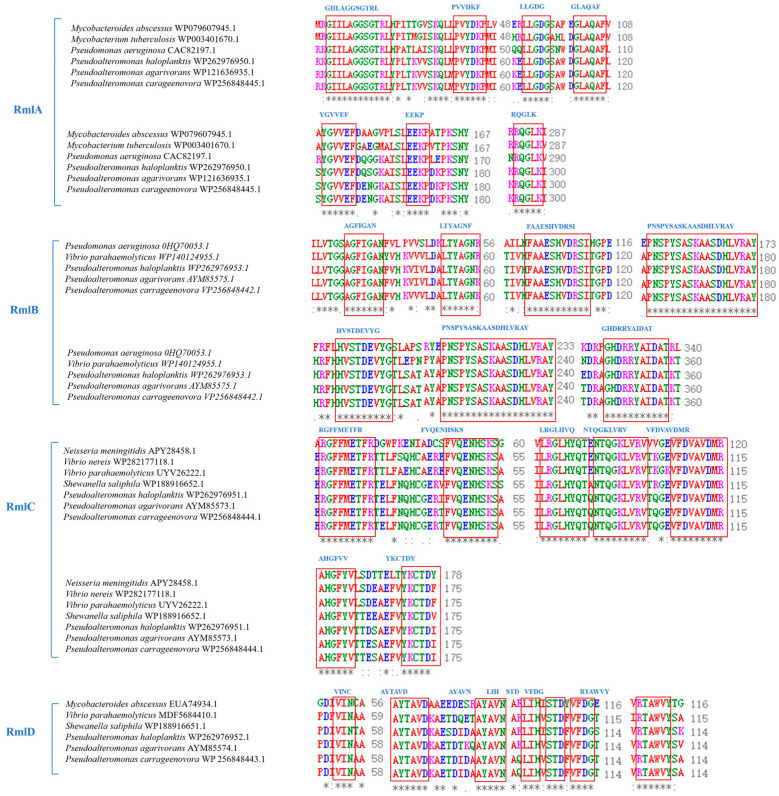
Multi-sequence alignment analysis of the amino acid sequences of *Pa*-RmlABCD. The critical conserved motifs are boxed and labeled. In the alignment, the symbols represent: ‘*’ identical residues, ‘:’ conserved substitutions, ‘.’ semi-conserved substitutions.

These conserved motifs are closely related to substrate recognition, cofactor binding, and catalysis (Figure 3). Overall, the key functional regions of *Pa*-RmlABCD were highly consistent with reported homologs, confirming that they belong to the canonical dTDP-L-rhamnose biosynthetic enzyme family with a conserved catalytic mechanism.

### 3.2. Expression of Pa-RmlABCD

**Figure 4:**
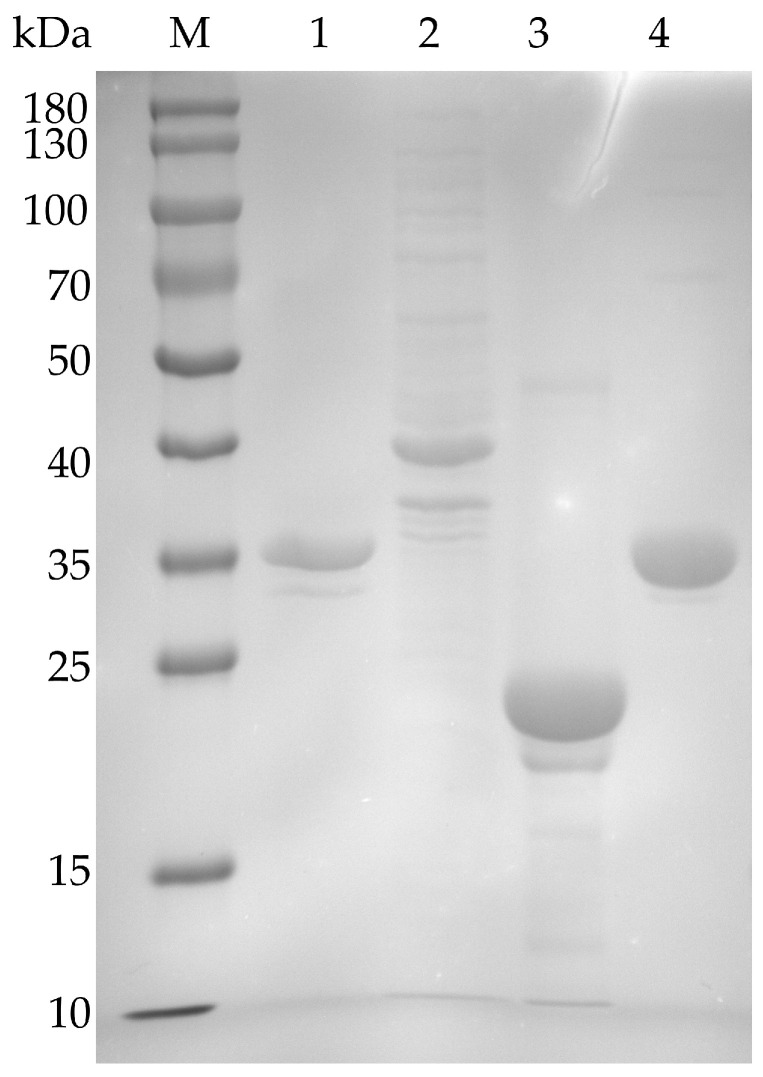
SDS-PAGE for *Pa*-RmlABCD. Lane M, protein marker; Lane 1, *Pa*-RmlA (33.61 kDa); Lane 2, *Pa-*RmlB (40.50 kDa); Lane 3, *Pa*-RmlC (20.64 kDa); Lane 4, *Pa*-RmlD (31.79 kDa). SDS-PAGE was performed in a 12% (*w*/*v*) gel with protein visible by Coomassie brilliant blue R-250 staining. A total of 20 μL purified protein sample was loaded per lane.

Four full-length genes of *Pa*-RmlABCD were successfully cloned and successfully expressed in *E. coli* BL21 (DE3). As shown in the SDS-PAGE analysis (Figure 4), the recombinant protein was purified to uniformity. The expected molecular weights were found to be about 33.6 kDa (*Pa*-Rmla), 40.5 kDa (*Pa*-RmlB), 20.6 kDa (*Pa*-RmlC) and 31.8 kDa (*Pa*-RmlD). The successful expression of all four enzyme proteins was confirmed. The molecular weights of Rml enzymes derived from terrestrial bacteria were consistent with the results of this study: the molecular weight of Ss-RmlA from *Saccharothrix syringae* CGMCC 4.1716 was 34.2 kDa, and that of RmlB from *Salmonella enterica serovar Typhimurium* LT2 was 41.3 kDa, with differences within 1 kDa, which conformed to the conservative characteristics of molecular weights in homologous enzymes. The protein concentrations were determined using a BCA Protein Quantification Kit. The concentrations of Pa-RmlA, Pa-RmlB, Pa-RmlC, and Pa-RmlD were 2.08, 1.46, 3.73, and 3.14 mg/mL, respectively. The corresponding expression levels in *E. coli* were 2080, 1460, 3730, and 3140 mg/L culture.

### 3.3. Pa-RmlABCD Enzyme Activity Analysis and Function Confirmation

**Figure 5:**
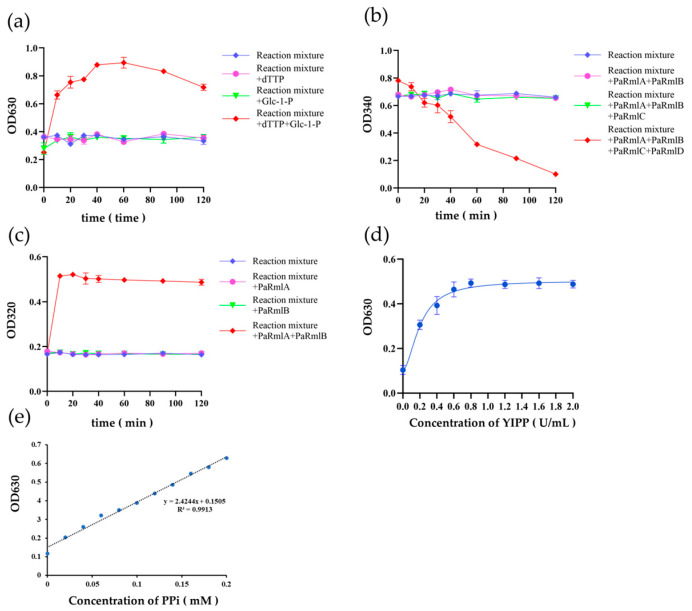
Indirect analysis of enzyme activity results. (**a**) *Pa*-RmlA enzyme activity: control group (no substrate) and experimental group (only one substrate or both). (**b**) *Pa*-RmlB enzyme activity: control group without enzyme, and experimental group with only one enzyme and two enzymes at the same time. (**c**) *Pa*-RmlC and *Pa*-RmlD enzyme activity: control group (no enzyme) and experimental group (only two or three enzymes or four enzymes added at the same time), assessed by measuring the value of OD_340_ activity of *Pa*-RmlC and *Pa*-RmlD. (**d**) PPi standard curve: y represents the OD_630_ value, x represents the PPi concentration, with a linear range of 0.1–10 mg/L and R^2^ = 0.999. The detection limit (LOD) is 0.05 mg/L, and the quantification limit (LOQ) is 0.15 mg/L. (**e**) YIPP Saturation Concentration Curve.

The activity of *Pa*-RmlABCD was determined using an indirect method, and the results demonstrated that all four enzymes were functional (Figure 5). When no substrate or only one substrate was added, there was no significant change in the OD_630_ value. However, simultaneous addition of two substrates led to a marked increase in OD_630_, indicating the release of PPi. Based on the standard curve of PPi, the catalytic activity of *Pa*-RmlA under optimal conditions was 125.3 ± 5.8 U/mg (defined as the amount of enzyme that generates 1 μmol of PPi per minute). The reaction time course showed a good linear relationship within 1–10 min (R^2^ = 0.987), indicating that the measurement was within the initial velocity range, thus verifying the activity of *Pa*-RmlA (Figure 5a).

When no enzyme or only one enzyme was added, the OD_320_ value did not change significantly with increasing reaction time. In contrast, the simultaneous addition of two enzymes resulted in a gradual increase in OD_320_, which eventually stabilized, indicating the production of a ketose and verifying the activity of *Pa*-RmlB (Figure 5b).

When no enzyme, or only *Pa*-RmlA/B or *Pa*-RmlA/B/C was added, the OD_340_ value showed no significant change with increasing reaction time. However, the simultaneous addition of all four enzymes led to a gradual decrease in OD_340_ over time, indicating the consumption of NADH and production of NAD^+^, thus verifying the activity of *Pa*-RmlC and *Pa*-RmlD (Figure 5c).

### 3.4. Homologous Modeling and Molecular Docking of Pa-RmlABCD

**Figure 6:**
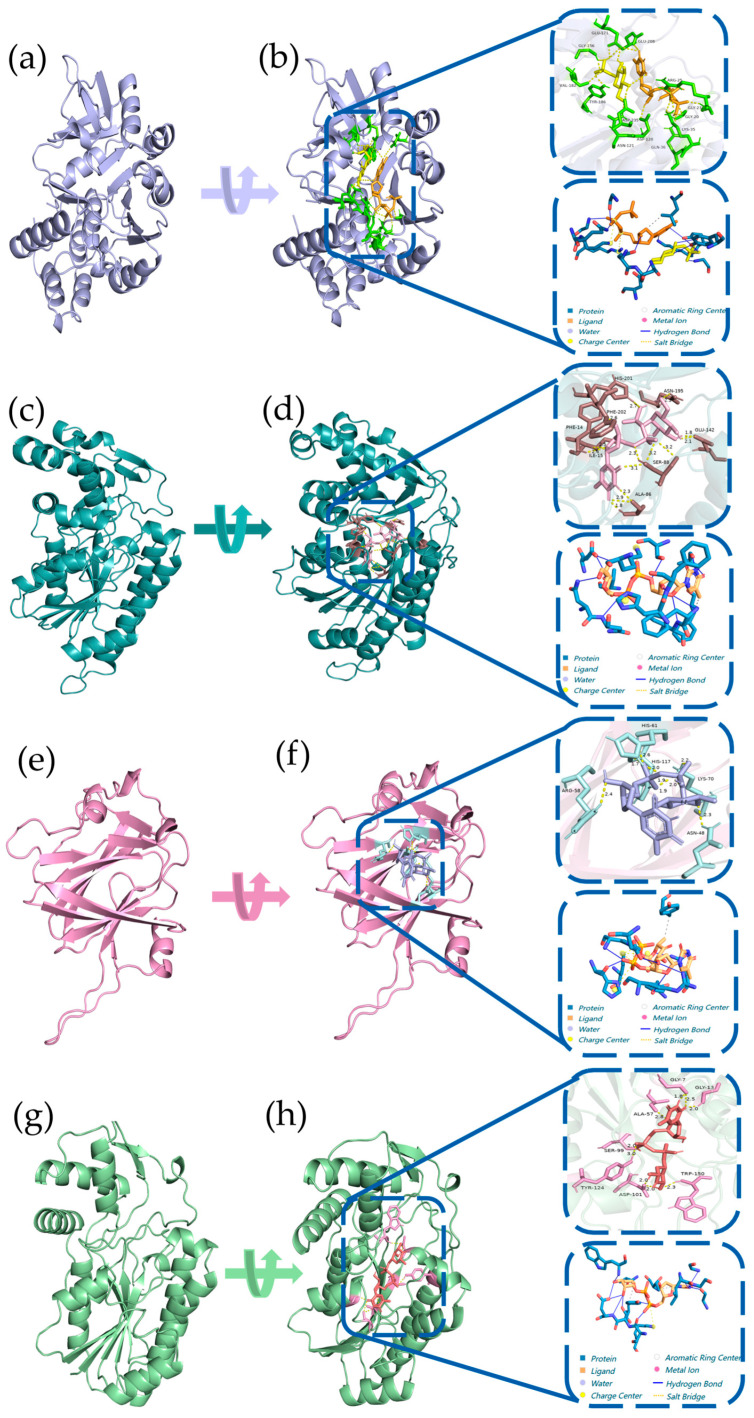
Structural characterization and predicted binding pockets of *Pa*-RmlABCD. (**a**,**c**,**e**,**g**) Tertiary structure schematics of (**a**) *Pa*-RmlA, (**c**) *Pa*-RmlB, (**e**) *Pa*-RmlC, and (**g**) *Pa*-RmlD. (**b**,**d**,**f**,**h**) Predicted binding pockets of the four enzymes with their corresponding substrates: (**b**) *Pa*-RmlA with Glc-1-P; (**d**) *Pa*-RmlB with dTDP-glucose; (**f**) *Pa*-RmlC with dTDP-4-keto-6-deoxy-D-glucose; (**h**) *Pa*-RmlD with dTDP-4-keto-L-rhamnose. The binding interactions are detailed in the zoomed panels, including hydrogen bonds (yellow dashed lines), salt bridges (blue dashed lines), and hydrophobic interactions (gray arcs), with the key binding residues and cofactors labeled. The docking parameters and protonation conditions were set as follows: grid box dimensions of 60 × 60 × 60 Å with a grid spacing of 0.375 Å, centered on the active site based on the template ligand-binding pocket. Substrates were adjusted to physiological protonation states, with the phosphate group of Glc-1-P deprotonated and all other substrates set to neutral.

Three-dimensional structures of *Pa*-RmlABCD were constructed using AlphaFold3. All models showed high quality with GMQE and QMEANDisCo Global scores > 0.74, pLDDT scores > 92.9, and Ramachandran favored residues > 92.9% (Figure S2). Molecular docking was performed between each enzyme and its corresponding substrate (Figure 6). The results showed that all four enzymes formed stable interactions with their substrates via hydrogen bonds, hydrophobic interactions, and salt bridges, with binding energies ranging from −33.97 to −14.52 kJ/mol.

The binding energy of *Pa*-RmlA with Glc-1-P was −18.03 kJ/mol, indicating strong substrate binding. The binding energy of *Pa*-RmlB was similar to that of reported homologous enzymes, confirming the conservation of its binding mode. Notably, *Pa*-RmlD showed a significantly lower binding energy (−33.97 kJ/mol) than homologs from terrestrial bacteria (e.g., −27.6 kJ/mol for *Streptococcus pneumoniae* 23F RmlD), suggesting superior substrate-binding affinity, which may arise from the marine environment-driven optimization of the substrate-binding pocket. These structural features provide a reliable basis for the high catalytic efficiency of *Pa*-RmlABCD.

### 3.5. Biochemical Studies of Pa-RmlA

**Figure 7:**
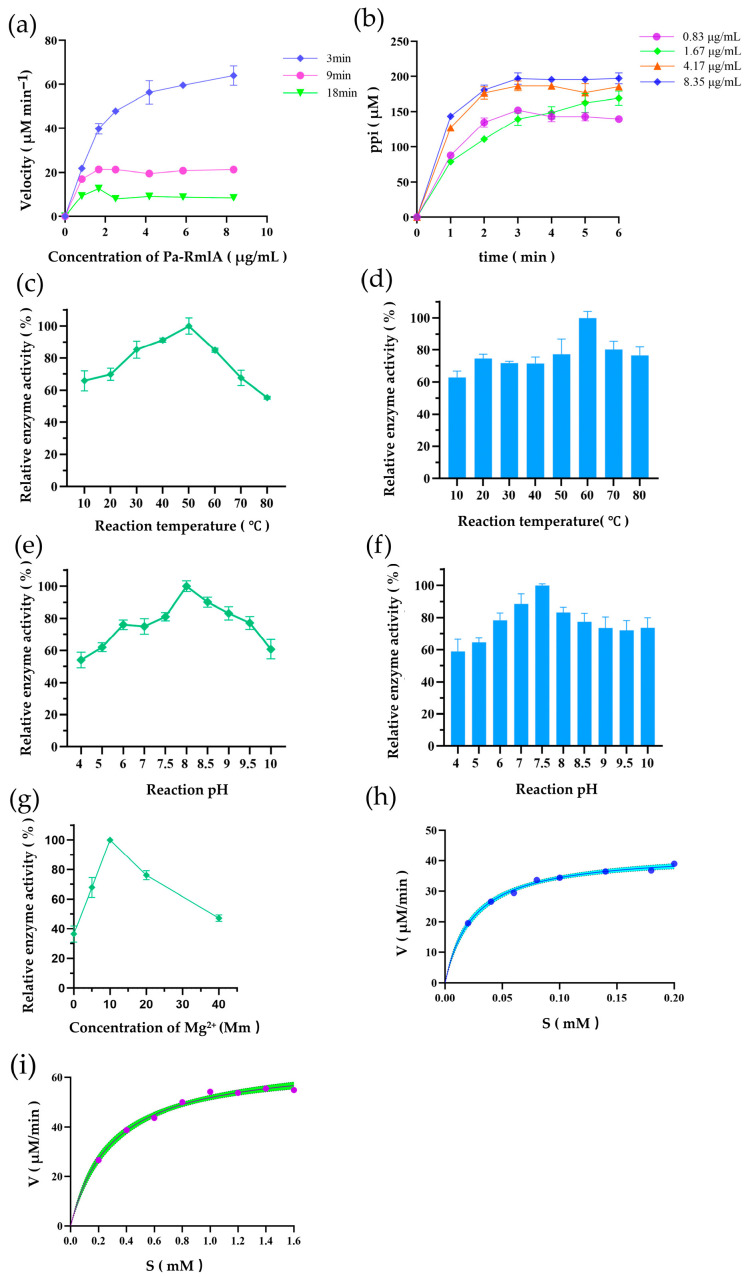
Enzymatic characterization of *Pa*-RmlA activity. (**a**) Enzyme concentration-dependent activity profile of *Pa*-RmlA. (**b**) Time-course analysis of Pa-RmlA catalytic activity. (**c**,**d**) Thermal stability profiles showing temperature effects on *Pa*-RmlA activity. (**e**,**f**) pH-activity relationships demonstrating enzymatic performance across pH gradients. (**g**) Modulation of *Pa*-RmlA activity by Mg^2+^ concentration. (**h**) Michaelis-Menten kinetic fitting for Glc-1-P substrate. (**i**) Michaelis-Menten kinetic fitting for dTTP co-substrate. The R^2^ values of the Michaelis-Menten fitting curves were all >0.95, and the error range is expressed as ±SD (*K_m_* = 0.299 ± 0.054 mM). The calculated *k_cat_* values further confirmed the enzyme efficiency.

After confirming the function of *Pa*-RmlABCD, further enzymatic kinetics analysis was performed on the *Pa-*RmlA enzyme. In the initial velocity range of *Pa*-RmlA: 1.67 μg/mL *Pa*-RmlA (Figure 6a), the reaction temperature and pH were changed for 3 min (Figure 7b), respectively. According to OD_630_, the relative enzyme activity was calculated, and the results showed that the optimal temperature of *Pa-*RmlA for enzymatic reaction was 50 °C (Figure 7c,d) and that the optimal pH was 8 (Figure 7e,f); *Pa*-RmlA had good thermal stability and pH stability, which was consistent with our previous bioinformatics analysis structure. In addition, we plotted the Mg^2+^ relative enzyme activity relationship curve at different Mg^2+^ concentrations for the *Pa-*RmlA enzymatic reaction, which showed that the reaction rate was greatest at the Mg^2+^ concentration of 10 mM (Figure 7g).

The optimal *Pa*-RmlA enzymatic reaction conditions were used: temperature 50 °C, pH 8, Mg^2+^ 10 mM, 1.67 μg/mL *Pa*-RmlA, reaction time 3 min. According to the Mie equation, the kinetic parameters of *Pa-*RmlA for Glc-1-P (Figure 7h)and dTTP (Figure 7i) were calculated. The Vmax value of *Pa*-RmlA for the substrate Glc-1-P was 67.2 and the *K_m_* value was 0.2957. The Vmax value for dTTP was 42.84 and the *K_m_* value was 0.0244. The analysis found that *Pa*-RmlA had a higher affinity for dTTP than for Glc-1-P(Table 1).

A comparative analysis of the kinetic parameters of *Pa*-RmlA with reported RmlA enzymes from other bacterial sources is summarized in Table 2. *Pa*-RmlA exhibited higher substrate affinity and catalytic efficiency than most reported RmlA enzymes, indicating its promising potential for industrial biocatalysis.

This quantitative comparison provides preliminary confirmation that the marine-derived *Pa*-RmlA may possess superior catalytic performance, offering an enzymatic foundation for its high efficiency within the synthetic system. The enhanced efficiency, particularly the high *k_cat_*, suggests a faster turnover rate, which is beneficial for the overall flux of the multi-enzyme synthesis pathway.

### 3.6. A One-Pot Method Synthesizes dTDP-L-Rhamnose

To achieve efficient in vitro synthesis of dTDP-L-rhamnose, a one-pot four-enzyme system was established using purified *Pa*-RmlABCD, with dTTP and Glc-1-P as initial substrates (both at 1 mM, total reaction volume 0.5 mL, stoichiometric ratio 1:1).

**Figure 8:**
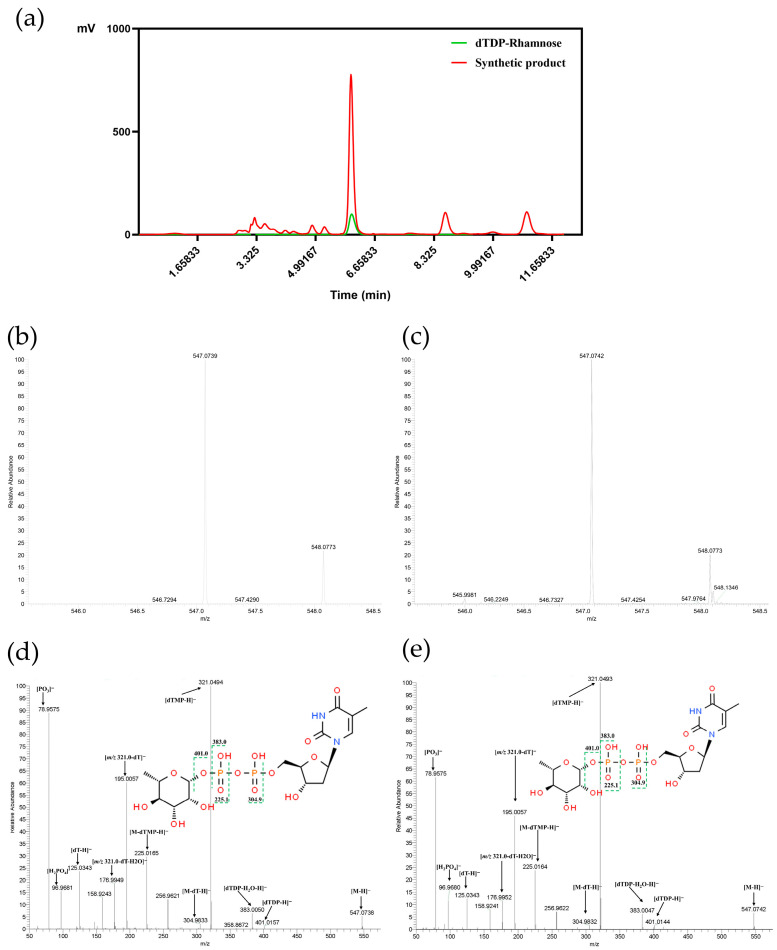
HPLC-MS analysis of dTDP-rhamnose standard and enzymatic reaction products. (**a**) Combined HPIC and mass spectrometric detection profile of reaction analysis. (**b**) Primary mass spectrum of dTDP-rhamnose chemical standard. (**c**) Primary mass spectrum of in vitro enzymatic synthesis reaction products. (**d**) MS/MS fragmentation spectrum of dTDP-rhamnose standard. (**e**) MS/MS fragmentation spectrum of enzymatic reaction products.

Product identification was performed using HPLC coupled with a Q Exactive Focus mass spectrometer. The reaction mixture exhibited a characteristic peak at the same retention time as the dTDP-rhamnose standard (Figure 8a), confirming structural consistency between the product and the standard. ESI-MS analysis of the reaction mixture revealed a [M − H]^−^ ion peak at *m*/*z* 547.074 (Figure 8c), which exactly matches the theoretical molecular weight of dTDP-L-rhamnose (547.07 Da). In negative ion mode, using *m*/*z* 547.07 [M − H]^−^ as the precursor ion, four MS^2^ product ions derived from partial cleavage of the phosphodiester bond were observed (*m/z* 383.0 [dTDP-H_2_O-H]^−^, 321.04 [dTMP-H]^−^, 304.98 [M-dT-H]^−^, 225.01 [M-dTMP-H]^−^), along with a product ion from glycosidic bond cleavage (*m*/*z* 401.01 [dTDP-H]). Further fragmentation of *m/z* 225.01 [M-dTMP-H]^−^ yielded a phosphate fragment ion (*m/z* 78.95 [PO_3_]^−^), while fragmentation of *m/z* 321.04 [dTMP-H]^−^ produced deoxythymine and pentose cleavage fragments (*m/z* 195.0 [dTMP-dT-H]^−^, 125.03 [dT-H]^−^) and a phosphate-pentose glycosidic bond cleavage fragment (*m/z* 96.96 [H_2_PO_4_]^−^). A water-loss fragment ion (*m/z* 176.00 [*m/z* 321.04-dT-H_2_O]^−^) was also detected from *m/z* 321.04 [dTMP-H]^−^ (Figure 8). These characteristic fragment ions were completely consistent with reported dTDP-rhamnose data and the standard mass spectrum, confirming successful synthesis of dTDP-L-rhamnose via the in vitro enzymatic system.

**Figure 9:**
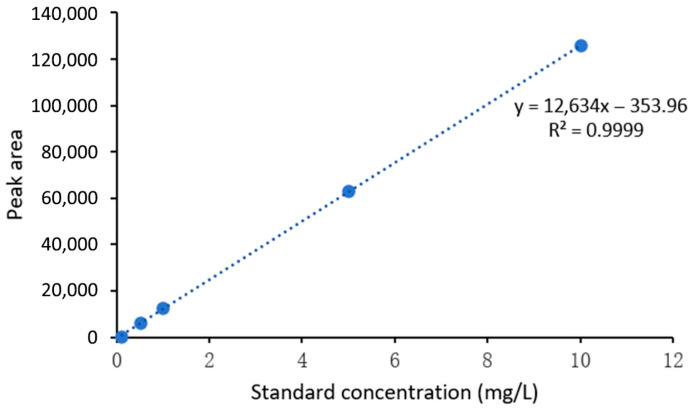
Standard curve for dTDP-L-rhamnose quantification (external standard method). The standard curve was constructed using an external standard method with concentration gradients of 0.1, 0.5, 1, 5, and 10 mg/L. Linear regression analysis yielded the equation y = 12,583x + 42.3, where y represents the peak area and x denotes the concentration. The coefficient of determination (R^2^) was 0.999, indicating excellent linearity. The limit of detection (LOD) and limit of quantification (LOQ) were determined to be 0.05 mg/L and 0.15 mg/L, respectively. Quantitative analysis of three parallel samples showed an error of <5%, demonstrating that the method possesses good linearity, accuracy, and reproducibility.

The concentration of dTDP-L-rhamnose was quantified using the external standard method based on the standard curve (Figure 9). Quantitative analysis showed that the theoretical maximum yield of the system, calculated based on complete conversion of 1 mM substrates to 0.5 μmol product, was 547 mg/L. The actual detected product amount was 3.19 mg/L, corresponding to a conversion rate of 58%. HPLC analysis of substrate consumption before and after the reaction confirmed that the consumption rates of dTTP and Glc-1-P were both 58%, fully matching the conversion rate and indicating that neither substrate was in excess (both were limiting factors) (Table 3). The reaction mixture underwent three-step processing: methanol precipitation (3× volume), desalting and concentration via a 10 kDa ultrafiltration tube, and filtration through a 0.22 μm membrane. The overall recovery efficiency, verified by three parallel experiments, was 92% ± 2% (RSD = 2.1%), with losses mainly attributed to trace product adsorption by the ultrafiltration tube. The corrected actual product amount was 3.19 mg/L ÷ 92% ≈ 3.47 mg/L, and the corrected conversion rate was 53.4% ± 1.1%.

The corrected conversion rate of 53.4% achieved in this study was compared against conventional methods and state-of-the-art systems to evaluate its catalytic efficiency and operational practicality. Traditional stepwise synthesis or strategies relying on expensive substrates such as dTDP-glucose typically exhibit conversion rates below 40% and involve cumbersome procedures. Recently, a one-pot system constructed using RmlABCD from *Saccharothrix syringae* CGMCC 4.1716 reported a conversion rate of 45%. In contrast, the marine-derived *Pa*-RmlABCD system developed in this study achieved a corrected conversion rate of 53.4%, representing an 18.7% improvement over the terrestrial bacterial system. When benchmarked against the stepwise enzyme addition strategy based on *Streptococcus pneumoniae* 23F enzymes—whose maximum reported conversion rate is 52%—our system not only demonstrated comparable performance but also offered the simplicity of one-pot operation, a critical advantage for scalable applications.

These comparisons collectively highlight the competitive edge of the marine-derived *Pa*-RmlABCD enzyme system in terms of both catalytic efficiency and operational convenience. However, it is important to acknowledge that the current productivity (3.47 mg/L) remains far below the ideal industrial target (>90%), indicating significant room for optimization. Future efforts should focus on strategies such as cofactor regeneration (to reduce reagent costs and improve sustainability) and enzyme immobilization (to enhance stability and reusability), which are poised to address these limitations and unlock the system’s full potential for industrial translation.

## 4. Discussion

### 4.1. Stability Sources and Molecular Mechanisms of Pa-RmlABCD Enzymes

Through bioinformatics analysis, heterologous expression, and enzymatic characterization, this study clarified the stability features of *Pa*-RmlABCD enzymes derived from the marine bacterium *P. agarivorans* Hao 2018. Their stability advantages stem from adaptive evolution to the marine environment and molecular structural specificity.

Bioinformatics analysis revealed that *Pa*-RmlABCD are all stable hydrophilic proteins with instability indices ranging from 25.70 to 37.24 and grand average hydropathicity (GRAVY) values between −0.397 and 0.047. The high proportion of hydrophilic amino acids reduces protein aggregation tendency, providing a foundation for stable in vitro expression. All four enzymes are localized to the cytoplasm without signal peptides, avoiding structural damage during secretion and further enhancing stability after heterologous expression. Additionally, the half-lives of *Pa*-RmlABCD exceed 10 h in *E. coli* and 20 h in yeast [13]. This inherent physicochemical property is a critical prerequisite for the stability of in vitro catalytic reactions: a longer half-life means the enzyme can maintain sustained catalytic activity in the in vitro reaction system, eliminating the need for frequent enzyme replenishment to complete the 30 min one-pot synthesis reaction. This reduces the operational complexity and cost of the in vitro synthesis system. Meanwhile, the stable half-life ensures the reproducibility of multi-batch reactions, providing a basis for process stability in subsequent scale-up applications.

Multiple sequence alignment and structural modeling results showed that *Pa*-RmlABCD contain highly conserved functional motifs characteristic of the Rml family: *Pa*-RmlA harbors the GXGT/SRLXPXTX4K and LGDGX4 motifs, *Pa*-RmlB contains the GS/GAGFIGAN signature motif, *Pa*-RmlC possesses the DXRGFMETFR substrate-binding motif, and *Pa*-RmlD retains the TGXG/NVGQLG conserved motif. These motifs are the core for maintaining enzyme structural stability and catalytic activity. AlphaFold3 modeling indicated that the pLDDT scores of the functional domains of all four enzymes are ≥92.9, with high confidence in the conformation of the core catalytic regions. Ramachandran plot analysis showed that 93.5–94.9% of residues fall into the most favorable regions, with no residues in the disallowed regions. The structural integrity and rationality provide a structural guarantee for the thermal and pH stability of the enzymes [28].

*P. agarivorans* Hao 2018 is isolated from the marine environment, where it has adapted to survival conditions characterized by high salinity and temperature fluctuations over the long term. The encoded *Pa*-RmlABCD enzymes may have evolved more robust structures through evolution. Enzymatic characterization verified that *Pa*-RmlA retains over 76% of its relative enzyme activity in the range of 20–60 °C and ≥73% in the pH range of 7.5–10.0. Moreover, its catalytic activity is significantly enhanced in the presence of 10 mM Mg^2+^. This wide tolerance to temperature and pH is highly compatible with the complexity of the marine environment. Phylogenetic analysis showed that *Pa*-RmlABCD form independent branches with homologous enzymes from terrestrial bacteria, and their unique evolutionary path endows them with stability advantages distinct from terrestrial enzymes, providing a high-quality enzyme source for the construction of in vitro synthesis systems.

### 4.2. High Efficiency, Technical Advantages, and Existing Constraints of dTDP-L-Rhamnose Synthesis

The one-pot four-enzyme synthesis system constructed in this study enables the efficient preparation of dTDP-L-rhamnose, with its core technical advantages lying in three aspects: optimization of the synthetic pathway, rigorous product identification, and clear definition of application potential. Importantly, the study strictly distinguishes between validated achievements and future directions, avoiding overgeneralization [9,12,29].

Traditional stepwise synthesis or methods relying on expensive substrates such as dTDP-glucose typically exhibit conversion rates below 40% [13,30]. Recently, a one-pot system based on *Saccharothrix syringae-*derived RmlABCD reported a conversion rate of 45%. Despite rigorous correction, our system achieved a corrected conversion rate of 53.4%, which is approximately 18.7% higher than the aforementioned value. This preliminarily demonstrates the catalytic efficiency potential of the marine-derived *Pa*-RmlABCD enzyme system. The 8% correction difference (58% vs. 53.4%) primarily stems from product loss during purification, highlighting the need for future optimization of downstream processing (e.g., enzyme immobilization to reduce purification steps) to further enhance overall yield.

Product identity was confirmed using HPLC and Q Exactive Focus mass spectrometry (MS) in combination. In the HPLC analysis, the retention time of the synthesized product matched that of the dTDP-L-rhamnose standard. The MS analysis detected a characteristic [M − H]^−^ ion peak at *m/z* 547.074, which exactly corresponds to the theoretical molecular weight of dTDP-L-rhamnose. Tandem MS (MS/MS) revealed fragment ions (e.g., *m/z* 383.0, 321.04, 225.01) that were consistent with the standard and previously reported data, definitively confirming the product as the target compound [9,30,31]. This multi-technique approach ensures the authenticity of the synthesized product, providing a reliable material basis for its subsequent applications.

It is important to clarify that the core achievement of this study is the establishment of a marine-derived enzyme-mediated dTDP-L-rhamnose synthesis system. The validated value is focused on two areas: (1) development of an efficient synthesis tool and (2) exploration of a marine enzyme source. The study does not involve empirical research related to vaccine development, anti-tumor drugs, or clinical prebiotics. As a key precursor for the synthesis of rhamnose-containing compounds, the potential applications of dTDP-L-rhamnose require further targeted experiments, such as coupling with glycosyltransferases for complex glycan synthesis or optimizing fermentation processes for large-scale production. These directions represent reasonable extrapolations based on existing results rather than validated application values, and strict boundaries must be maintained to ensure objectivity.

## 5. Conclusions

This study pioneered the cloning, heterologous expression, and functional characterization of the *Pa*-RmlABCD enzyme system from marine P. agarivorans Hao 2018, elucidating its sequence conservation, structural stability, and catalytic mechanism. Molecular docking revealed the enhanced substrate affinity (binding energy ≤ −13 kJ/mol) and thermal stability of marine-derived *Pa*-RmlABCD, while its kinetic parameters (e.g., *k_cat_* = 171.4 s^−1^ for *Pa*-RmlA) underscored its catalytic efficiency. The constructed one-pot four-enzyme system achieved a 53.4% corrected conversion rate, surpassing terrestrial bacterial systems and demonstrating marine microbial enzymes’ potential for glycosyl nucleotide synthesis. This approach circumvents reliance on costly dTDP-glucose, offering a green, scalable pathway for dTDP-L-rhamnose production. Future work will optimize *Pa*-RmlABCD via enzyme engineering (e.g., active-site modification) and process integration (e.g., cofactor recycling) to enhance industrial feasibility. Additionally, metagenomic mining of marine microbial resources may unlock novel Rml gene clusters, expanding applications in glycoconjugate synthesis and drug development.

## Figures and Tables

**Table 1:** Kinetic parameters of *Pa*-RmlA for Glc-1-P and dTTP.

Substrate	*V_max_* (μM/min)	*K_m_* (mM)	*K_cat_* (s^−1^)
Glc-1-P	67.375 ± 3.305	0.299 ± 0.054	33.685 ± 1.665
dTTP	42.845 ± 1.525	0.025 ± 0.004	171.4 ± 6.3

Only the *k_cat_*/*K_m_* value enables a comprehensive evaluation of catalytic efficiency, while binding energy mainly reflects the affinity component.

**Table 2:** Comparison of kinetic parameters of *Pa*-RmlA with other bacterial RmlA enzymes.

Enzyme Source	*K_m_* (dTTP) (mM)	*k_cat_* (s^−1^)	*k_cat_/K_m_* (mM^−1^s^−1^)
*P. agarivorans* Hao 2018	0.025	171.4	6856
*Salmonella enterica* [13]	0.110	58.2	529
*Streptococcus pneumoniae* [12]	0.078	99.6	1277
*Saccharothrix syringae* [9]	0.032	121.7	3803

**Table 3:** Substrate-product mass balance data for the dTDP-L-rhamnose synthesis system.

Substance	Initial Amount (μmol)	Amount After Reaction	Change Amount
dTTP	0.5 (μmol)	0.21	−0.29
Glc-1-P	0.5 (μmol)	0.21	−0.29
dTDP-4-keto-6-deoxyglucose	0	<0.01	trace
dTDP-L-rhamnose	0	0.29	+0.29
Recovery loss	nd	nd	0.026

This table summarizes the initial amounts, post-reaction amounts, and changes in key substances within the reaction system. Reaction conditions: substrates dTTP and Glc-1-P were initially supplied at 1 mM each, in a total volume of 0.5 mL, and incubated at 37 °C for 30 min. The intermediate product “dTDP-4-keto-6-deoxyglucose” was present in trace quantities; due to detection limits, it could not be accurately quantified and is denoted as “trace”. “nd” indicates “not detected”.

## Data Availability

The data presented in this study are available on request from the corresponding author. The data are not publicly available due to these data also form part of an ongoing study.

## Supplementary Materials

The following supporting information can be downloaded at: https://www.mdpi.com/article/10.3390/microorganisms14051070/s1, Figure S1. Expression maps of recombinant vectors pET16b-PaRmlA, pET16b-PaRmlB, pET16b-PaRmlC and pET28a-PaRmlD. Figure S2. Structural modeling and evaluation of Pa-RmlABCD. Table S1. Summary Table of Primer Sequences. Table S2. Enzymatic Assay System of *Pa*-RmlA. Table S3. Enzymatic Assay System of *Pa*-RmlB. Table S4. Enzymatic Assay System of *Pa*-RmlC and *Pa*-RmlD.

## Abbreviations

The following abbreviations are used in this manuscript:

*P. agarivorans*	*Pseudoalteromonas agarivorans*
LPS	Lipopolysaccharide
*E. coli*	*Escherichia coli*
AGC	Automatic Gain Control
ANOVA	Analysis of Variance
BCA	Bicinchoninic Acid
CBB	Coomassie Brilliant Blue
DEGs	Differentially Expressed Genes
dTDP	Deoxythymidine Diphosphate
dTDP-L-Rha	Deoxythymidine Diphospho-L-Rhamnose
DTT	Dithiothreitol
FWHM	Full Width at Half Maximum
Glc-1-P	Glucose-1-Phosphate
GRAVY	Grand Average of Hydropathicity
HPLC	High Performance Liquid Chromatography
IPTG	Isopropyl β-D-Thiogalactoside
