# Supplementary material for: Characterization of RmlABCD Enzymes from Marine Bacteria and Efficient Synthesis of dTDP-L-Rhamnose
Source: Microorganisms. 2026 May 9;14(5):1070. doi: 10.3390/microorganisms14051070 (PMC13210114; doi:10.3390/microorganisms14051070)
Supplement: Supplementary file 1 [file microorganisms-14-01070-s001.zip › microorganisms-4240565-supplementary.pdf]

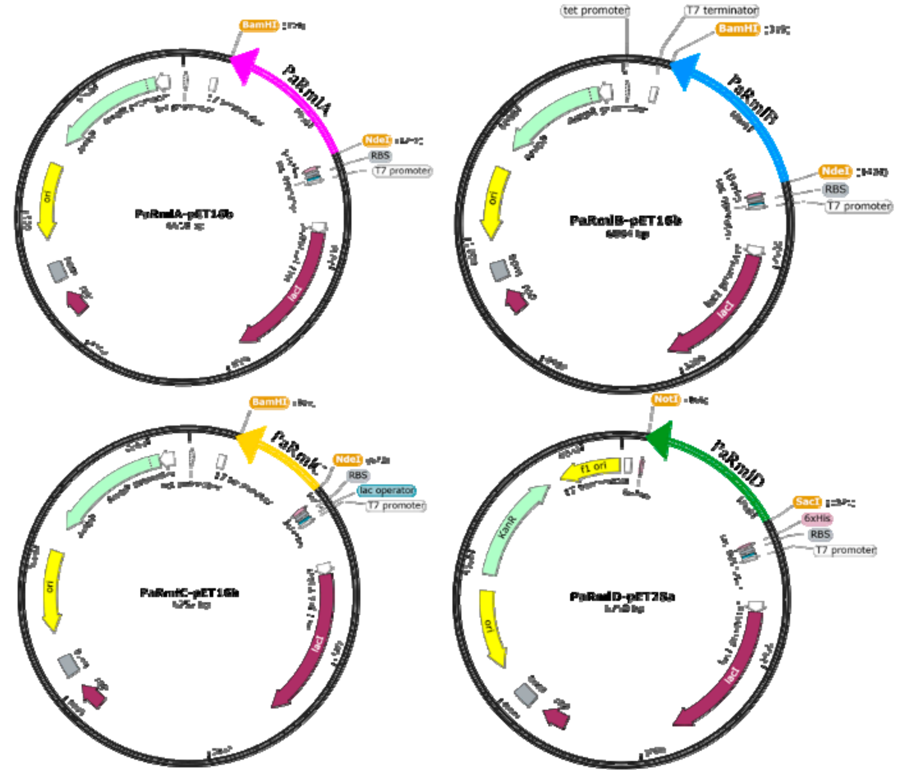

Figure S1. Expression maps of recombinant vectors pET16b-PaRmlA, pET16b-PaRmlB, pET16b-PaRmlC and pET28a-PaRmlD

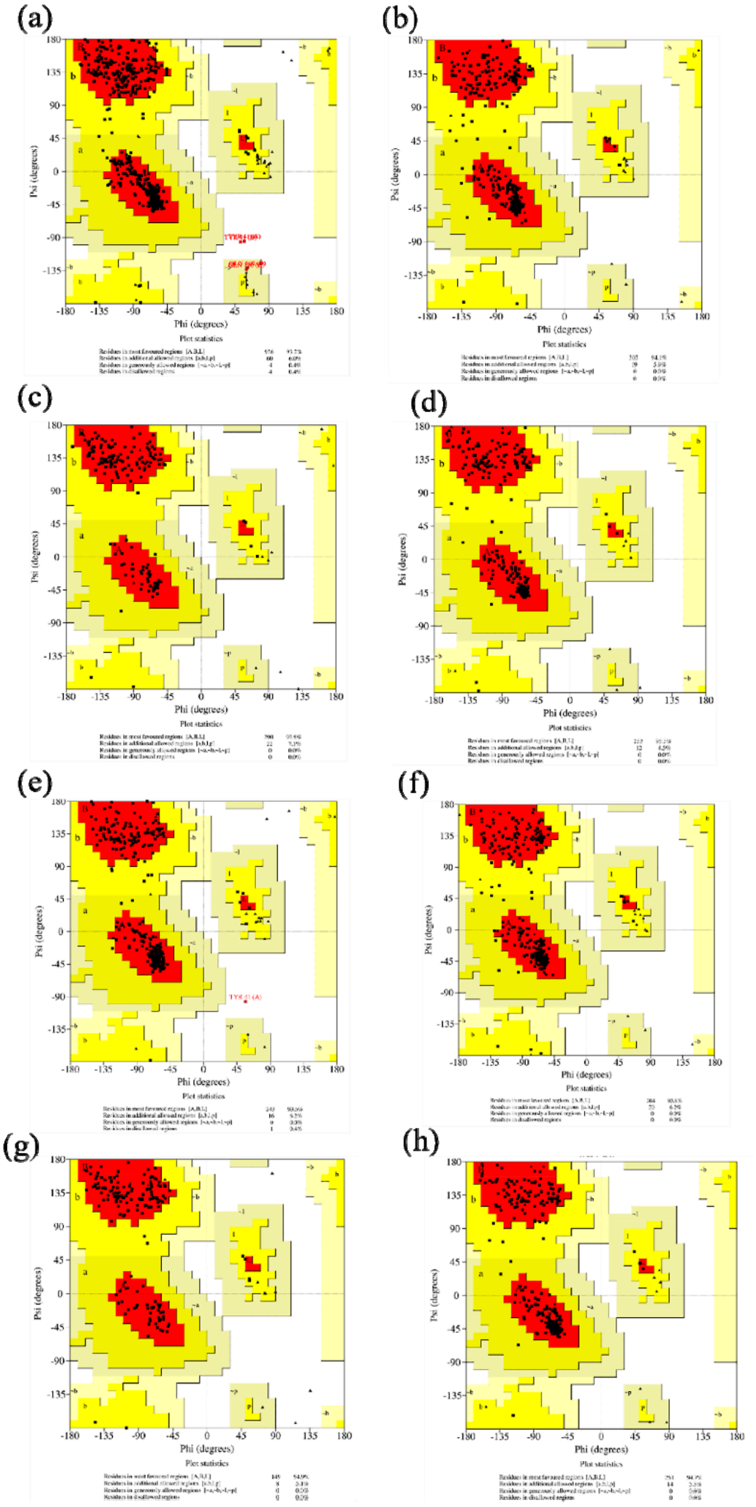

Figure S2. Structural modeling and evaluation of Pa-RmlABCD.(a–d) Ramachandran plot analysis of homologous models of Pa-RmlA, Pa-RmlB, Pa-RmlC and Pa-RmlD generated by SWISS-MODEL.(e–h) Ramachandran plot analysis of the structural models predicted by AlphaFold 3. AlphaFold 3 modeling was performed with default parameters under standard model confidence mode, and the maximum number of generated models was set to 5. In each plot, the color gradient from dark to bright represents the favored region, allowed region,

generously allowed region, and disallowed region, respectively. Triangles, squares and dots denote amino acid residues located in different regions.According to previous studies, the GMQE and QMEANDisCo Global scores both range from 0 to 1: 0–0.6 indicates poor model quality with obvious structural deviations (such as backbone distortion and unreasonable side-chain conformation), and the model should be used with caution; 0.6–0.8 represents moderate model quality, in which key functional domains are likely reliable while overall structural verification is still required; scores ⩾ 0.8 indicate high-quality models with structures close to the native state, which can be directly used for functional analysis .Molecular docking was carried out using AutoDock V, with 50 independent docking runs performed for each ligand. The binding energy values are presented as mean ± standard deviation (e.g., the binding energy between Pa-RmlA and glucose-1-phosphate was −18.03 ± 0.67 kJ/mol, n = 50). Cluster analysis showed that the proportion of the dominant conformation was over 80% with an RMSD value less than 2.0 Å, indicating high robustness of the docking results.

Table S1. Summary Table of Primer Sequences.

**Primer Name**	**Primer Sequence (5′ to 3′)**
Pa-RmlA-F	CGCATATGATGTATTCGTTAAAAGCCCCAG
Pa-RmlA-R	CGCGGATCCTTAAAAAACAGTTTGATCTAAAAG
Pa-RmlB-F	GCGCGCCATATGGTGAATTTTATAGAAACCGAT
Pa-RmlB-R	CGGGATCCTTAAAACGTAGGTGCTTTTGTA
Pa-RmlC-F	CGAGCTCATGAAAGTATTAGTAACAGGTAAAAATGGA
Pa-RmlC-R	ATGCGGCCGCTTAAGCTTGCGCCTTTAACTCTATT
Pa-RmlD-F	GCGCGCCATATGATGACTGTAAAAAATCTATT
Pa-RmlD-R	CGCGGATCCTTAAGAGTTATAAGAGCCGT

Table S2. Enzymatic Assay System of *Pa*-RmlA.

**Reagents**	**0**	**1**	**2**	**3**
1M Tris-HCl（pH 7.5）	2.5μL	2.5μL	2.5μL	2.5μL
50% (v/v) glycerol	10μL	10μL	10μL	10μL
100 mM $MgCl_2$	2.5μL	2.5μL	2.5μL	2.5μL
10 mM DTT	5μL	5μL	5μL	5μL
10 mM dTTP	—	—	1μL	1μL
10 mM Glc-1-P	—	5μL	—	5μL
0.01U/ml YIPP	1μL	1μL	1μL	1μL
Pa-RmlA	1μL	1μL	1μL	1μL
RNase-free ddH_2O	28μL	23μL	27μL	22μL

* Group 0 served as the blank control (lacking both dTTP and Glc-1-P), Group 1 contained only Glc-1-P, Group 2 contained only dTTP, and Group 3 was the dual-substrate group (containing both dTTP and Glc-1-P). All reagent volumes were in μL, with a total reaction volume of 50 μL. Reactions were incubated at 37°C for 10–120 minutes, after which 50 μL of malachite green chromogenic solution (composed of 0.35‰ (w/v) malachite green, 0.5‰ (v/v) Triton X-100, and 2.5‰ (w/v) ammonium molybdate in 0.7 mol/L hydrochloric acid) was added to terminate the reaction. The mixture was incubated at 37°C for 5 minutes before measuring the absorbance at 630 nm (OD_{630} nm). The catalytic activity of PaRmlA was verified by comparing the OD_{630} nm values across groups.

Table S3. Enzymatic Assay System of *Pa*-RmlB.

Reagents	0	1	2	3
1M Tris-HCl（pH 7.5）	2.5μL	2.5μL	2.5μL	2.5μL
10 mM DTT	1μL	1μL	1μL	1μL
50% (v/v) glycerol	10μL	10μL	10μL	10μL
100 mM $MgCl_2$	2.5μL	2.5μL	2.5μL	2.5μL
10 mM Glc-1-P	—	5μL	—	5μL
NAD^+	1μL	1μL	1μL	1μL
Pa-RmlA (50 μg/mL)	—	2μL	—	2μL
Pa-RmlB (50 μg/mL)	—	—	2μL	2μL
RNase-free ddH_2O	28μL	23μL	27μL	22μL

* Group 0 served as the blank control (without enzyme), Group 1 contained only *Pa*-RmlA, Group 2 contained only *Pa*-RmlB, and Group 3 was the dual-enzyme group (containing both *Pa*-RmlA and *Pa*-RmlB). All reagent volumes were in μL, with a total reaction volume of 50 μL and an enzyme solution concentration of 50 μg/mL for each enzyme. Reactions were incubated at 30°C for 10 minutes, after which 150 μL of 100 mM sodium hydroxide solution was added to terminate the reaction. The mixture was allowed to stand for 15 minutes before measuring the absorbance at 320 nm (OD_{320} nm). *Pa*-RmlA first catalyzes the reaction between dTTP and Glc-1-P to generate dTDP-glucose, which is then dehydrated by *Pa*-RmlB to form dTDP-4-keto-6-deoxy-D-glucose (the ketone group exhibits characteristic absorption at 320 nm).

Table S4. Enzymatic Assay System of *Pa*-RmlC and *Pa*-RmlD.

Reagents	0	1	2	3
1M Tris-HCl（pH 7.5）	2.5 μL	2.5 μL	2.5 μL	2.5 μL
10 mM DTT	1 μL	1 μL	1 μL	1 μL
50% (v/v) glycerol	10 μL	10 μL	10 μL	10 μL
100 mM $MgCl_2$	2.5 μL	2.5 μL	2.5 μL	2.5 μL
10 mM Glc-1-P	5 μL	5 μL	5 μL	5 μL
NAD^+	1 μL	1 μL	1 μL	1 μL
Pa-RmlA (50 μg/mL)	—	2 μL	2 μL	2 μL
Pa-RmlB (50 μg/mL)	—	2 μL	2 μL	2 μL
Pa-RmlC（50 μg/mL）	—	—	2 μL	2 μL
Pa-RmlD（50 μg/mL）	—	—	—	2 μL
RNase-free ddH_2O	38 μL	34 μL	32 μL	30 μL

* Group 0 served as the blank control (without enzyme), Group 1 contained *Pa*-RmlA+B, Group 2 included *Pa*-RmlA+B+C, and Group 3 comprised *Pa*-RmlA+B+C+D. All reagent volumes were in μL, with a total reaction volume of 50 μL and an enzyme solution concentration of 50 μg/mL for each enzyme. Reactions were incubated at 37°C for 10–120 minutes, and absorbance at 340 nm (OD_{340} nm) was measured directly
